# Berberine ameliorates testosterone-induced benign prostate hyperplasia in rats

**DOI:** 10.1186/s12906-021-03472-2

**Published:** 2021-12-20

**Authors:** Ehsan Shabani, Heibatullah Kalantari, Mojtaba Kalantar, Mehdi Goudarzi, Esrafil Mansouri, Hadi Kalantar

**Affiliations:** 1 Toxicology Research Center, Medical Basic Sciences Research Institute, Ahvaz Jundishapur University of Medical Sciences, Ahvaz, Iran; 2 Department of Toxicology, School of Pharmacy, Ahvaz Jundishapur University of Medical Sciences, Ahvaz, Iran; 3 Shoushtar Faculty of Medical Sciences, Shoushtar, Iran; 4 Medicinal Plant Research Center, Ahvaz Jundishapur University of Medical Sciences, Ahvaz, Iran; 5 Cellular and Molecular Research Center, Medical Basic Sciences Research Institute, Ahvaz Jundishapur University of Medical Sciences, Ahvaz, Iran

**Keywords:** Berberine, BPH, Testosterone, Oxidative stress, Inflammation, Rat

## Abstract

**Introduction:**

Benign prostatic hyperplasia (BPH) is a major urologic problem that mostly develops in older males. Oxidative stress and inflammation influence the occurrence of BPH. Berberine (BBR) is a natural ingredient that has antioxidant and anti-inflammatory properties. The current research aims at examining the effects of BBR on testosterone-stimulated BPH in rats.

**Methods:**

Animals were randomly categorized to six groups. In the control group, normal saline and olive oil were injected as the vehicle. BPH group: received testosterone (3 mg/kg, subcutaneous, 28 days), BPH + BBR groups; received BBR (25 and 50 mg/kg, p.o, 28 days), BPH + finasteride groups: received finasteride (1 mg/kg, p.o, 28 days), BBR (50 mg/kg, p.o, alone) was administered for subjects in the BBR group. On the 29th day, after anesthesia, cervical dislocation was used to kill the subjects. Serum concentration of testosterone and dihydrotestosterone was measured and prostate tissues were excised and used for biochemical, inflammation, and histological analysis.

**Results:**

BBR prevented increased serum concentrations of testosterone and dihydrotestosterone. BBR considerably reduced BPH-stimulated oxidative stress and inflammation through preventing the rise in lipid peroxidation and nitrite concentration and declined the accumulations of pro-inflammatory cytokines (e.g. interleukin 1β and tumor necrosis factor α) and declining the depletion rate of GSH and the function of catalase and superoxide dismutase. Histopathological investigations reported that administration of BBR could suppress testosterone-stimulated BPH.

**Conclusion:**

This study demonstrated that BBR could significantly prevent the development of BPH in rats.

## Introduction

Benign prostatic hyperplasia (BPH) is an urological problem in elderly males. After the age of 40, nearly one-third of old males develop BPH. After the 8^th^ decade of life, its prevalence reaches about 80% [1, 2]. This disease is described as a noncancerous prostate disorder resulting in enlargement of the prostate, and causes lower urinary tract symptoms (LUTS) [1]. LUTS contains the enhanced frequency of urination, unnecessary contraction of the bladder and nocturia. These presentations are correlated with enhanced risk of urinary tract blockage, not emptying the bladder and urinary infection [3]. It is worth noting that the exact etiology of BPH is not clear yet, but, disturbing androgen/estrogen balance as well as over secretion of growth factors has a key role in BPH [4, 5]. In addition, oxidative stress has made a crucial contribution to BPH [6, 7]. Imbalanced generation of reactive oxygen species (ROS) and anti-oxidants parameters results in oxidative stress, which may damage the main parts of tissues like proteins, DNA, and mRNA [8]. Oxidative stress may indicate the increased risk of cellular proliferation, which causes hyperplastic growth in prostatic tissue [9]. Furthermore, it may stimulate nuclear factor kappa B (NF-κB) that may result in regulation of inflammatory as well as cellular proliferation mechanisms [10]. Recent studies have found a relationship between inflammation of prostatic and unregulated stromal and epithelial cell proliferation via various mechanisms that contain hormonal alternations and secretion of different inflammatory cytokines [11–14]. Medications that are used for managing BPH contain 5α-reductase inhibitors (such as finasteride) and α1- receptor antagonists (such as tamsulosin) and surgical treatments [11, 15]. Nevertheless, their application is restricted because of complications with surgical prostatectomy and drug side effects, like gynecomastia, dizziness, headache, erectile dysfunction, loss of libido [16–18]. Regarding this issue, it is of crucial importance to find ingredients that can prevent BPH development (with no considerable side-effect) [12]. It's well-proved that several natural compounds can be used as therapeutic agents with few adverse effects to treat human disorders [19, 20].

Berberine (BBR, an isoquinoline alkaloid) is a biologically active component of traditional Chinese plants available in many plants such as *Ranunculaceae, Coptis sp. Rutaceae, Berberis sp.* The main feature of BBR is its high safety [21, 22]. Recent studies have indicated that BBR has multiple pharmacological properties, such as antioxidant [23, 24], anti-inflammatory [25, 26], antidiabetic [27], anticancer [27], antibacterial [28], antihyperlipidaemic [29], antidepressant, anti-anxiety, anti-psychosis, anti-amnesia [30, 31]. Furthermore, BBR is effective in preventing testicular damage [24, 32], cardiotoxicity [33], hepatotoxicity [34] and Alzheimer 's disease [35]. Nevertheless, no research has investigated its ameliorative properties over testosterone-stimulated BPH in rats. Hence, the current study aims to examine whether BBR can inhibit testosterone-stimulated prostatic hyperplasia in rats.

## Methods

### Chemicals

BBR (CAS: 633–65-8), testosterone (CAS Number: 58–22-0), finasteride (CAS Number: 98319–26-7), and other chemicals and reagents prepared from Sigma-Aldrich (Germany).

### Animal

Forty-eight male Wistar rats (220–250 g) were obtained from the animal house of Ahvaz Jundishapur University of Medical Sciences (AJUMS). Then, for seven days, the rats were placed in a 12:12 h sleep–wake cycle in ideal states (normal moisture, 65 ± 5%; temperature, 23 ± 1 °C). The current research is confirmed by the Ethics Committee of Ahvaz Jundishapur University of Medical Sciences (**Ethics code:** IR.AJUMS.ABHC.REC. 1397.069). This study was in accordance with the standards set forth in the eighth edition of “Guide for the Care and Use of Laboratory Animals” (grants.nih.gov/grants/olaw/guide-for-the-care-and-use-of-laboratory-animals_prepub.pdf published by the National Academy of Sciences, The National Academies Press, Washington, D.C.). It is in line with the ARRIVE (Animal Research: Reporting of in Vivo Experiments) guidelines about using and coring of research animals.

### Study design

The animals were randomly separated into six categories (eight in each, n = 8).

Group 1 (negative control group) received normal saline (5 mL/kg p.o., the vehicle of BBR and finasteride) and olive oil (1 mL/kg, s.c., the vehicle of testosterone) for 28 days.

Group 2 (BPH group) received testosterone (3 mg/kg, s.c.) for 28 days [36, 37].

Group 3 (positive control group), was given finasteride (1 mg/kg, p.o., daily) [38] 2 h prior to administering testosterone (3 mg/kg, s.c.) for 28 days.

Groups 4 and 5, were given BBR at doses of (25 & 50 mg/kg, p.o., daily) [25, 39, 40] respectively, 2 h before administration of testosterone (3 mg/kg) for 28 days.

Group 6 received BBR at a dose of (50 mg/kg, p.o. alone).

On the first and twenty-ninth days, the animals were weighed and, after sacrificing the animals, prostates were removed and measured. Then, the ratio of prostate weight to body weight was determined in all groups.

### Sample collection

At 24 h following the eventual therapy, all subjects were anesthetized by xylazine/ketamine (8/80 mg/kg; i.p). Then, blood samples collected from the heart were centrifuged at (1500 g) for 10 min in order to collect serum. The obtained samples were kept at − 70 °C. Then, cervical dislocation was used to scarify the subjects in order to take samples were prostates. The samples were rinsed using saline and divided into two symmetrical parts. Afterward, a cool phosphate buffer (0.01 M, pH 7.4) and a homogenizer were used to homogenize the first part as a 10% (weight/volume), centrifuged at (2000 g) for 15 min. Then, the supernatant was aliquoted and kept at − 70 °C until its application to identify biochemical and inflammation analysis. A buffer (formalin (10%)) was used to fix the second part for histopathological analysis.

### Serum Concentration of testosterone and dihydrotestosterone (DHT)

A testosterone and DHT ELISA kits (MyBioSource, USA) were used to estimate the serum levels of testosterone and DHT.

### Measurement of protein content

By applying the Biuret method (Ernest, 1996), we measured the protein content. It is worth noting that bovine serum albumin (BSA) was administered as the main criterion. So that 2.9 mL of normal saline and 3 mL of Biuret reagent were mixed in 0.1 ml of the sample. Afterward, the content was put at the temperature room for 10 min in order to measure the absorbance rate at 540 nm by an ELISA reader (RayBiotech, Canada). A BSA standard curve was used to measure the protein concentration [41]

### Measurement of malondialdehyde (MDA)

Lipid peroxidation was computed using estimating colorimetric of thiobarbituric acid-reactive substance (TBARS) following the reaction of malondialdehyde with thiobarbituric acid. In summary, 500 µl of tissue supernatant was mixed with one ml trichloroacetic acid (TCA) (20%). Then, for 10 min, it was centrifuged (2000 g). One ml of the supernatant was mixed with 0.5 ml of 0.67% thiobarbituric acid (TBA) and heated for 30 min in a boiling water bath, then cooled and the absorbance was determined by an ELISA reader (RayBiotech, Canada) at 532 nm [42, 43].

### Measurement of Nitric oxide (NO)

Griess assay [44, 45] was used to identify NO level, so that 100 µl of the sample was mixed with 100 µl acidic Griess reagent and the absorbance was determined by an ELISA reader (RayBiotech, Canada) at 540 nm.

### Measurement of the glutathione level (GSH)

We computed GSH level by Ellman,s technique [46–48]. In summary, supernatant 1 mL was blended with 1 mL of 4% sulfosalicylic acid, then it was centrifuged at (1200 g) for a quarter-hour at 4 °C. Then, 2.7 mL of 0.1 M phosphate buffer (pH 7.4) and 0.2 mL of 5,5-dithiobis 2-nitrobenzoic acid (DTNB) (40 mg/10 mL of 0.1 M phosphate buffer, pH 7.4) were mixed. Afterward, the yellow color was investigated instantly at 412 nm by an ELISA reader (RayBiotech, Canada).

### Measurement of the catalase (CAT) activity

By a technique that measures the decomposition of hydrogen peroxide (H_2_O_2_), the function of CAT was evaluated [49, 50]. In summary, the sample contained 1.95 mL of phosphate buffer (0.05 M, pH 7), 1 mL of hydrogen peroxide (0.019 M), and 0.05 mL supernatant. Afterward, the absorbance was determined immediately at 240 nm by an ELISA reader (RayBiotech, Canada).

### Measuring superoxide dismutase (SOD) and glutathione peroxidase (GPx) functions

The functions of SOD and GPx were measured by commercial kits based on protocols approved by the manufactures (Zellbio, Germany).

### Measurement of the proinflammatory cytokines

Interleukin 1β (IL-1 β) and tumor necrosis factor α (TNF- α) levels were measured through ELISA and commercial kits (Ray Biotech, Canada).

### Histopathological analysis

The fixed ventral lobe of prostatic tissue embedded in paraffin wax was separated in 5 μm thick parts by a slide microtome. Then, the tissues were stained with hematoxylin and eosin and examined under a light microscope with magnification of 150x. The volume and width of epithelial cells and architectural changes in the ventral lobe of prostate tissue were evaluated [51–53].

### Data analysis

The findings are described using mean ± SD. The significance of difference in the different groups was evaluated by a one-way ANOVA and Tukey post hoc tests. Outputs have been evaluated as significant at *p* < *0.05*. Graph Pad Prism was used to analyze the data.

## Results

**Fig. 1:**
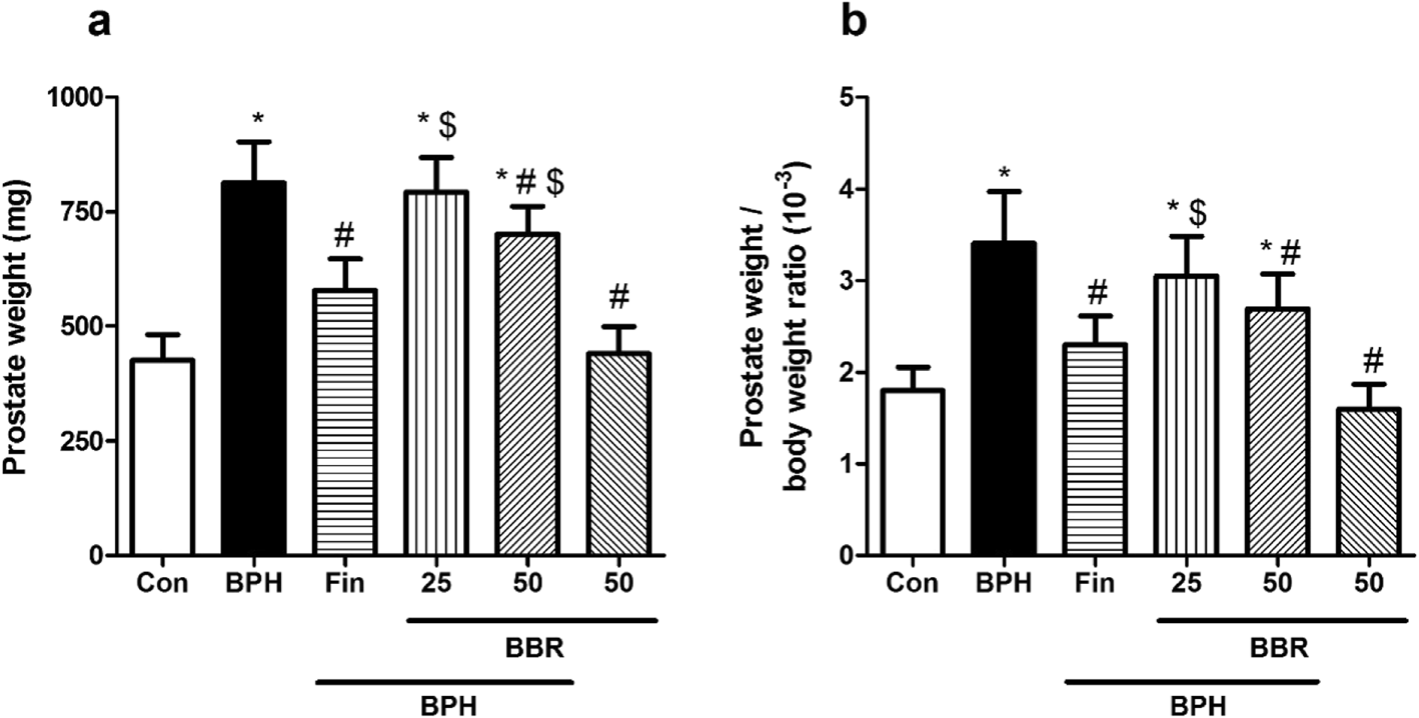
Influence on prostate weight and prostate weight/body weight ratio. Values are mean ± SD (n = 8). One-way ANOVA and Tukey’s tests were used for comparisons. * Significant difference in comparison with the control group. *(*p* < *0.05*). # Significant difference in comparison with the BPH group. #(*p* < *0.05*). $ Significant difference in comparison with the Fin group. $(*p* < *0.05*). *Con* Control, *BPH* Benign prostatic hyperplasia, *Fin* Finasteride, *BBR* Berberine

Influence on prostate weight and prostate weight/body weight ratio.Fig. 1a-b, reveals that injecting testosterone to rats (BPH group) caused a significantly increase in the prostate weight and prostate weight/body weight ratio than the control group (*p* < *0.05*). Furthermore, berberine-treated (at dose 50 mg/kg) as well as finasteride significantly reduced the prostate weight and prostate weight/body weight ratio than the BPH group (*p* < *0.05*). The BBR (50 mg/kg, alone) did not change in the prostate weight and prostate weight/body weight ratio in rats than the control group.

### Influence on testosterone and DHT

**Fig. 2:**
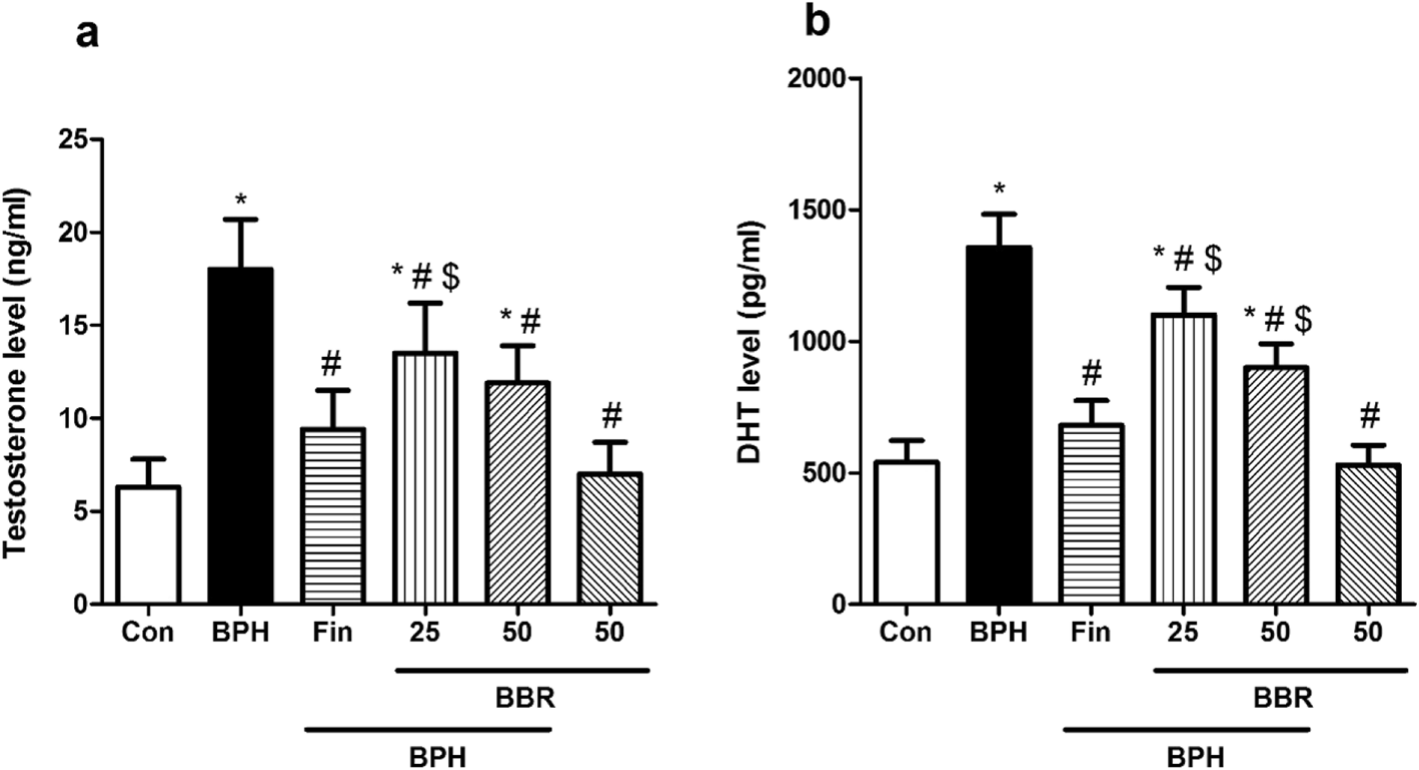
Effects of BBR on serum biochemical parameters. Values are mean ± SD (n = 8). One-way ANOVA and Tukey’s tests were used for comparisons. * Significant difference in comparison with the control group. *(*p* < *0.05*). # Significant difference in comparison with the BPH group. #(*p* < *0.05*). $ Significant difference in comparison with the Fin group. $(*p* < *0.05*). *Con* Control, *BPH* Benign prostatic hyperplasia, *Fin* Finasteride, *BBR* Berberine, *DHT* Dihydrotestosterone

The influence of BBR on serum markers is illustrated in Fig. 2a-b. A considerable rise in serum concentrations of testosterone and DHT in rats in the BPH group (*p* < *0.05*). Administration of BBR (at dose 25 and mg/kg) considerably declined serum concentration of testosterone and DHT than the BPH group (*p* < *0.05*). Like rats in BBR-treated group, those in the finasteride-treated group presented a significant decline in concentrations of testosterone and DHT than the BPH group (*p* < *0.05*). The BBR (50 mg/kg, alone) did not change in concentrations of testosterone and DHT in rats than the control group.

### Influence on MDA and NO levels

**Fig. 3:**
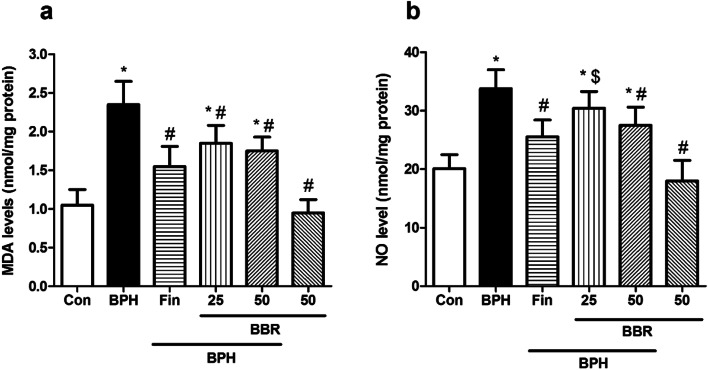
Effects of BBR on oxidative stress parameters. Values are mean ± SD (n = 8). One-way ANOVA ANOVA and Tukey’s tests were used for comparisons. * Significant difference in comparison with the control group. *(*p* < *0.05*). # Significant difference in comparison with the BPH group. #(*p* < *0.05*). $ Significant difference in comparison with the Fin group. $(*p* < *0.05*). *Con* Control, *BPH* Benign prostatic hyperplasia, *Fin* Finasteride, *BBR* Berberine, *MDA* Malondialdehyde, *NO* Nitric oxide

MDA and NO concentrations were considerably raised in the BPH rates than control rats (*p* < *0.05*). However, the BBR rats (at doses 25 and 50 mg/kg) considerably inhibited the rise in MDA concentration than the BPH group (*p* < *0.05*). Furthermore, BBR (at dose 50 mg/kg) considerably declined NO level than BPH group (*p* < *0.05*). Similarly, treatment of rats with finasteride significantly reduced concentrations of MDA and NO compared to the BPH group (*p* < *0.05*)*.* Moreover, oxidative stress parameters did not change after BBR (50 mg/kg, alone) administration, compared to the control rats. The influence of BBR on oxidative stress markers is illustrated in Fig. 3a-b.

### Influence on anti-oxidant markers

**Fig. 4:**
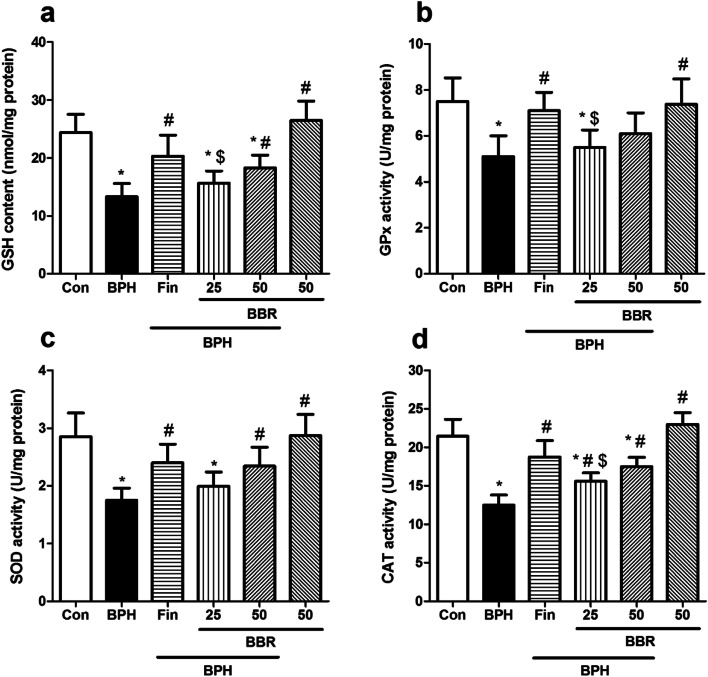
Effects of BBR on antioxidants markers. Values are mean ± SD (n = 8). One-way ANOVA and Tukey’s tests were used for comparisons. * Significant difference in comparison with the control group. *(*p* < *0.05*). # Significant difference in comparison with the BPH group. #(*p* < *0.05*). $ Significant difference in comparison with the Fin group. $(*p* < *0.05*). *Con* Control, *BPH* Benign prostatic hyperplasia, *Fin* Finasteride, *BBR* Berberine, *GSH* Glutathione, *GPx* Glutathione peroxidase, *SOD* Superoxide dismutase, *CAT* Catalase

As shown in Fig. 4a-d, in the BPH group, GSH content, and GPx, CAT, and SOD functions, significantly reduced than control rats (*p* < *0.05*). BBR treatment at 50 mg/kg resulted in a considerable rise in GSH level, SOD and CAT activity than BPH rats (*p* < *0.05*). Also, BBR enhanced GPx function than the BPH group but this increase was not significant. Furthermore, treating rats with finasteride significantly increased GSH content, as well as CAT*,* GPx and SOD (*p* < *0.05*) activities than the BPH group. Also, these antioxidant markers did not significantly change due to BBR (50 mg/kg, alone) administration than the control group.

### Influence on the IL-1β & TNF-α levels

**Fig. 5:**
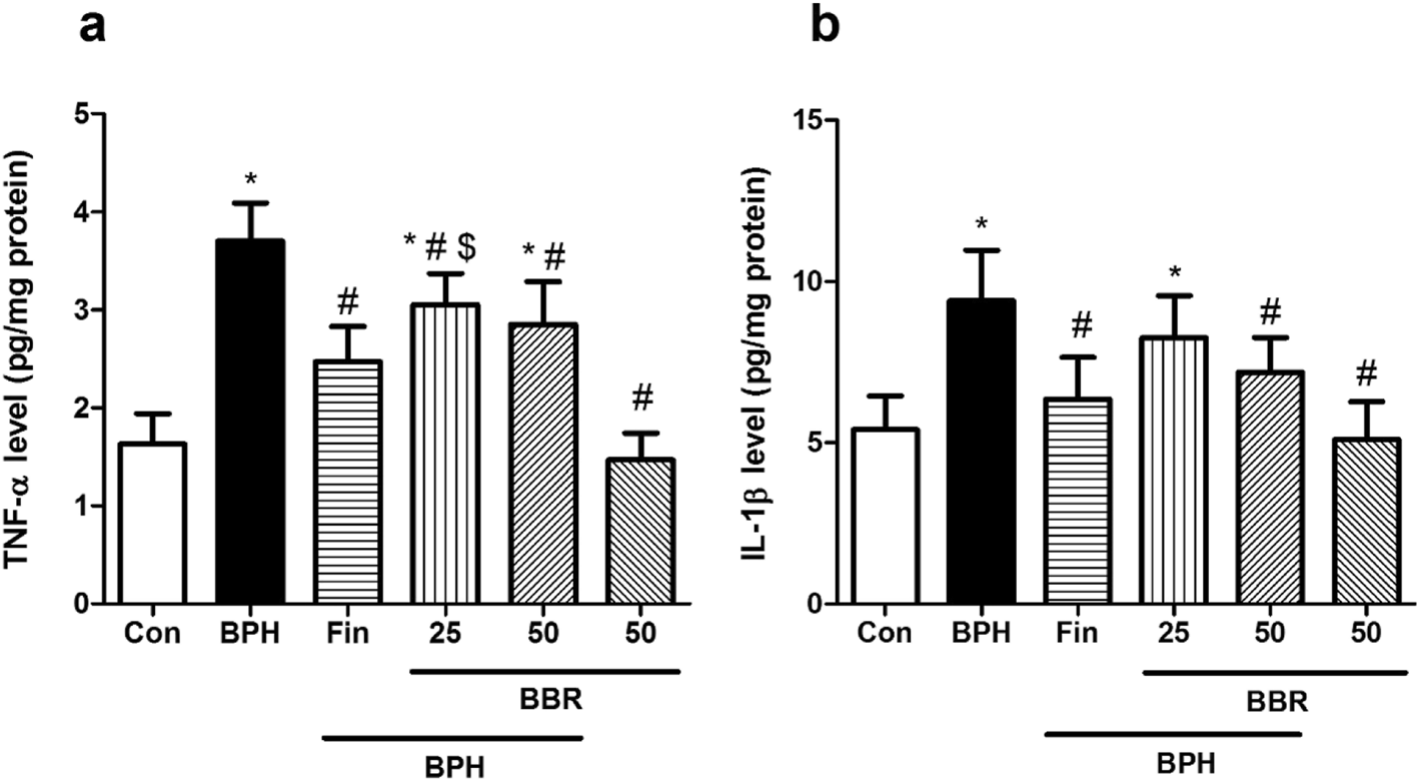
Effects of BBR on pro-inflammatory parameters. Values are mean ± SD (n = 8). One-way ANOVA and Tukey’s tests were used for comparisons. * Significant difference in comparison with the control group. *(*p* < *005*). # Significant difference in comparison with the BPH group. #(*p* < *0.05*)*. *$ Significant difference in comparison with the Fin group. $(*p* < *0.05*). *Con* Control, Benign prostatic hyperplasia, *Fin* Finasteride, *BBR* Berberine, IL-1 β: Interleukin 1β, TNF- α: Tumor necrosis factor α

Fig. 5a-b, reveals that injecting testosterone to rats (BPH group) caused a significantly increase in the concentrations of IL-1β and TNF-α (*p* < *0.05*). Furthermore, a considerable reduction in the levels of TNF-α (at dose 25 and 50 mg/kg, *p* < *0.05*) and IL-1β (at dose 50 mg/kg, *p* < *0.05*) in the BBR-treated were observed. Similarly, treatment of rats with finasteride significantly declined IL-1β and TNF-α concentrations than BPH group (*p* < *0.05*)*.* Also, these inflammatory markers did not significantly change due to BBR (50 mg/kg, alone) administration, compared to the control group.

### Histopathological investigation

**Fig. 6:**
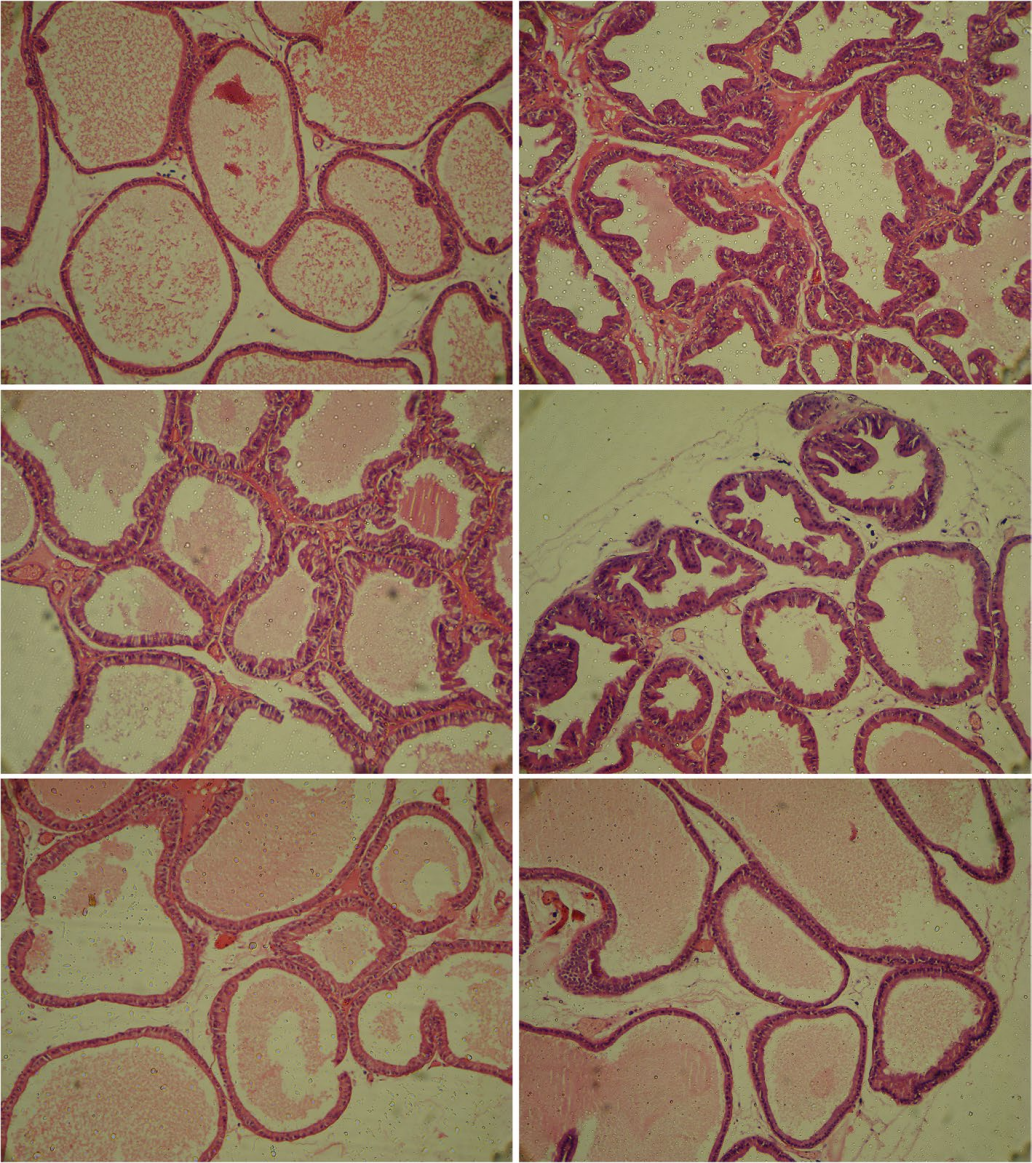
Histopathological observations (prostatic tissue stained with Hematoxylin & Eosin, magnification X150) showing effects of BBR and Fin on BPH. Prostate tissue of the BPH group showed morphological abnormalities in the prostate epithelial (as identified by enhanced thickness of epithelial and multiple unorganized layers (white arrow), and numerous intra-glandular lumen papillary folds (black arrow). BBR treatment improved prostatic hyperplasia. BBR (25 and 50 mg/kg) and Fin treatment improved BPH. *Con* Control, *BPH* Benign prostatic hyperplasia, *Fin* Finasteride *BBR* Berberine

As shown in Fig. 6, samples taken from the prostates of controls and those rats that received BBR (alone), presented typical morphological construction of epithelial cells, no histological alternation in prostate tissue. Concerning the shape and size, epithelial cells were cuboidal. On the other hand, prostate samples of the BPH group showed morphological abnormalities in the prostate epithelial (as identified by enhanced thickness of epithelial and multiple unorganized layers (white arrow), and numerous intra-glandular lumen papillary folds (black arrow). The treatment with finasteride and BBR (at doses of 25 and 50 mg/kg) presented some characteristics of prostate glandular hyperplasia, but a high decline in epithelial proliferation, papillary projections formation, and stromal spaces of the prostate in comparison to the BPH group.

## Discussion

According to the findings, BBR could decrease testosterone-stimulated benign prostate hyperplasia in rats. In the present study, testosterone significantly increased prostate weight, prostate weight/body weight ratio and histopathological alterations in the BPH group than to the control group. Administration of BBR significantly prevented the prostatic index and prostate weight, as well as finasteride effects, a drug now used to treat BPH. Histological examination of the prostate tissue of testosterone induced rats revealed thick epithelial layers, stromal proliferation and glandular hyperplasia. However, BPH animals that received BBR revealed a significant reduction in epithelial layer thickness and mild glandular hyperplasia, suggesting that BBR is an effective treatment for BPH. Testosterone and DHT are vital for usual functions of the prostate, growth, and occurrence of BPH [3, 4]. The 5α-reductase enzyme produces DHT from testosterone. Its affinity with androgen receptors (ARs) is three times higher than testosterone [4]. DHT can bind to ARs to initiate its biological properties including cell proliferation, survivorship, transcription of insulin-like growth factor 1 (IGF1), epidermal growth factor (EFG), fibroblast growth factor (FGFs), and so on [54, 55]. In agreement with our findings, the concentration of testosterone and DHT were considerably enhanced in the BPH rats [11, 12]. On the other hand, both BBR and finasteride had similar efficacy concerning normalizing concentration of testosterone and DHT in BPH rats, which indicates its high effect in addressing such conditions. According to the latest research, BBR prevents the secretion of testosterone by influencing aldo–keto reductase 1C3 in the 22Rv1 cell line [56]. There is a positive association between oxidative stress and BPH [49]. It's well-proved that androgens can increase prostatic cellular metabolism and proliferation, which causes an extensive spectrum of ROS and oxidative stress in conjunction with dampening antioxidant mechanisms in prostate tissue [57–60]. MDA is wields applied as a criterion of lipid peroxidation (LPO) [61]. Increased MDA may cause several problems, including prostate damage if not treated [11, 16, 62, 63]. In the present study, increased prostatic MDA in the BPH group was approved. Remarkably, BBR and finasteride considerably declined the MDA concentration that indicates the antioxidant properties of BBR and finasteride. Also, GSH is a major tool to keep redox homeostasis, which means preventing oxidative damage. GSH donates its electron to hydrogen peroxide (H_2_O_2_) to reduce it into H_2_O and O_2_ [64]. Moreover, antioxidant enzymes (CAT, SOD, GPx) scavenge ROS and prevent oxidative injury. CAT is a widely used antioxidant enzyme, mainly because it decomposes H_2_O_2_ into water and oxygen [65]. SOD transforms superoxide anion into hydrogen peroxide and oxygen, while GPx by removing H_2_O_2_ prevents its accumulation in the body [66]. In present research, a considerable decline in the antioxidant factors, GSH, SOD, GPx, and CAT enzymes in the BPH group has been observed. Treatment with BBR and finasteride significantly restored the GSH, CAT, SOD, (GPx by finasteride, only) and approved the antioxidant properties of the test ingredients. Previous studies have shown that BBR (as an anti-oxidant agent) can prevent damage to testicular tissue, reproductive function, and disruption of spermatogenesis through inhibiting the ROS/JAK2/NFκB pathway and reducing oxidative stress [32, 67].

NO is an unstable messenger molecule that is produced through inducible nitric oxide synthase (iNOS) and is involved in a wide range of physiological processes in the body [68]. In prostatic disorders, iNOS is the main variable that stimulates reactive nitrogen that may bring injury to the cells [69]. It's well-documented that NO is important for the correlation between inflammation and BPH [6, 66]. Prostatic inflammatory mediators like TNF-α, IL-1β, IL-2, -4,-6, 8, TGF-β, COX-2, and IFN-_ϒ_ are of crucial importance for BPH [12, 13, 66, 70, 71]. Experimentally, several studies have investigated the elements that prevent the secretion of inflammatory cytokines [66, 72]. In this research, testosterone considerably enhanced the concentrations of NO, TNF-α, and IL-1β in the prostate. Nevertheless, BBR reduced the concentration of these inflammatory agents, which is consistent with the literature [24, 25, 39, 40]. The protective properties of BBR against the enhanced secretion of pro-inflammatory cytokines may be associated with its antioxidant characteristics because oxidative stress induces their secretion via influencing the NF-κB [73].

## Conclusion

In summary, BBR treatment reduced concentrations of oxidative and inflammatory factors and enhanced the concentration of anti-oxidant variables in the prostate, which indicates that BBR can prevent BPH. Hence, it may be an appropriate herbal intervention.

## Data Availability

The datasets used and/or analyzed during the current study are available from the corresponding author on reasonable request.
